# Changes in Gene Transcription Induced by Hydrogen Peroxide Treatment of Verotoxin-Producing *Escherichia coli* O157:H7 and Non-O157 Serotypes on Romaine Lettuce

**DOI:** 10.3389/fmicb.2017.00477

**Published:** 2017-03-21

**Authors:** Gui-Ying Mei, Joshua Tang, Susan Bach, Magdalena Kostrzynska

**Affiliations:** ^1^Guelph Research and Development Centre, Agriculture and Agri-Food CanadaGuelph, ON, Canada; ^2^Summerland Research and Development Centre, Agriculture and Agri-Food CanadaSummerland, BC, Canada

**Keywords:** verotoxin-producing *Escherichia coli*, non-O157 serotypes, lettuce decontamination, hydrogen peroxide treatment, gene transcription

## Abstract

Disease outbreaks of verotoxin-producing *Escherichia coli* (VTEC) O157:H7 and non-O157 serotypes associated with leafy green vegetables are becoming a growing concern. A better understanding of the behavior of VTEC, particularly non-O157 serotypes, on lettuce under stress conditions is necessary for designing more effective control strategies. Hydrogen peroxide (H_2_O_2_) can be used as a sanitizer to reduce the microbial load in leafy green vegetables, particularly in fresh produce destined for the organic market. In this study, we tested the hypothesis that H_2_O_2_ treatment of contaminated lettuce affects in the same manner transcription of stress-associated and virulence genes in VTEC strains representing O157 and non-O157 serotypes. Six VTEC isolates representing serotypes O26:H11, O103:H2, O104:H4, O111:NM, O145:NM, and O157:H7 were included in this study. The results indicate that 50 mM H_2_O_2_ caused a population reduction of 2.4–2.8 log_10_ (compared to non-treated control samples) in all six VTEC strains present on romaine lettuce. Following the treatment, the transcription of genes related to oxidative stress (*oxyR* and *sodA*), general stress (*uspA* and *rpoS*), starvation (*phoA*), acid stress (*gadA*, *gadB*, and *gadW*), and virulence (*stx1A*, *stx2A*, and *fliC*) were dramatically downregulated in all six VTEC serotypes (*P* ≤ 0.05) compared to not treated control samples. Therefore, VTEC O157:H7 and non-O157 serotypes on lettuce showed similar survival rates and gene transcription profiles in response to 50 mM H_2_O_2_ treatment. Thus, the results derived from this study provide a basic understanding of the influence of H_2_O_2_ treatment on the survival and virulence of VTEC O157:H7 and non-O157 serotypes on lettuce.

## Introduction

Verotoxin-producing *Escherichia coli* (VTEC), also referred to as Shiga toxin (Stx)-producing *E. coli* (STEC), often cause life-threatening diseases, such as hemorrhagic colitis (HC) and hemolytic uremic syndrome (HUS) (Croxen et al., 2013; Karpman and Stahl, 2014; Karmali, 2017). Although VTEC serotype O157:H7 is commonly identified in human diseases, non-O157 serogroups have been increasingly associated with serious outbreaks and were recently responsible for more than 50% of STEC illness in U.S. (Luna-Gierke et al., 2014; Parsons et al., 2016; CDC, 2017). In 2015, the incidence of confirmed VTEC non-O157 infections was 40% higher than in 2012–2014 (Huang et al., 2016). More than 70% of infections linked to non-O157 VTEC were caused by serotypes O26, O45, O103, O111, O121, and O145 (termed the Top 6; Saito et al., 1998; Brooks et al., 2005; Folster et al., 2011; Bradley et al., 2012; Brown et al., 2012). In 2011, enteroaggregative *E. coli* O104:H4 caused the biggest outbreak in Germany. This strain produces Stx2 and is one of the most virulent strains of non-O157 VTEC (Buchholz et al., 2011; Zangari et al., 2013; Karmali, 2017).

Fresh leafy green vegetables are an important part of a healthy diet due to their richness in minerals, vitamins, and phytochemicals. However, leafy green vegetables can be contaminated by pathogenic bacteria such as VTEC during growth, harvesting, and transportation leading to subsequent illnesses and outbreaks (Wachtel et al., 2002; Solomon et al., 2003; Islam et al., 2004; Delaquis et al., 2007). A number of surveys have shown an increase in foodborne outbreaks linked to contaminated leafy green vegetables (Harris et al., 2003; Sivapalasingam et al., 2004; Rangel et al., 2005; Lynch et al., 2009; Painter et al., 2013; Herman et al., 2015). Although the contamination can be minimized by preventing produce exposure to sources of pathogenic bacteria, including contaminated water, soil, and animals, the occasional contamination of leafy green vegetables on farms by VTEC can still occur, which leads to contaminated produce entering the processing lines (Olaimat and Holley, 2012). Therefore, effectively reducing the contamination during processing is crucial to ensure the safety of fresh leafy green vegetables.

Chlorine has been widely used as a sanitizer to reduce the microbial load in fresh-cut vegetables (Beuchat et al., 1998). However, chlorine may react to form potentially carcinogenic or mutagenic products (Hurst, 1995). In addition, the by-products formed when sodium hypochlorite (NaOCl—another sanitizer commonly used in the fresh produce industry) reacts with organic compounds, have been shown to increase the risk of bladder cancer (Villanueva et al., 2004). Hydrogen peroxide (H_2_O_2_) is a potential alternative to chlorine treatment and breaks down to ecologically friendly water and oxygen (Linley et al., 2012). In addition, H_2_O_2_ has been approved for use in organic postharvest processing systems. As such, this sanitizer could be used for decontamination of fresh produce destined for organic market.

To date, several studies have been conducted on the gene expression in pure culture of *E. coli* O157:H7 under oxidative stress (Wang et al., 2009; Allen and Griffiths, 2012; Mei et al., 2015). In addition, using H_2_O_2_ as a sanitizer on fresh produce to reduce *E. coli* O157:H7 was investigated (Sapers et al., 2000; Huang et al., 2012). However, the survival and gene transcription of non-O157 VTEC serotypes under stress conditions on lettuce remain largely unknown. In this study, the behaviors of O157:H7 and non-O157 VTEC serotypes in response to H_2_O_2_ treatment on lettuce were evaluated.

## Materials and Methods

### Bacterial Strains and Growth Conditions

Six VTEC strains were tested in this study: *E. coli* O157:H7 (EDL933) (ATCC 700972), O26:H11 (EC20070549), O103:H2 (EC19970811), O104:H4 (NML#11-3088), O111:NM (EC20070546), and O145:NM (EC19970355). *E. coli* EDL933 was included in the present study because it is prototype O157:H7 strain. Serotypes O26, O103, O111, and O145 are predominant non-O157 serotypes worldwide, therefore strains representing these serotypes have been investigated in this study. In addition VTEC O104:H4 isolated from German outbreak was selected, because this strain is highly virulent and is linked to fresh produce outbreak. All the strains except EDL933 were of human origin. *E. coli* O157:H7 (EDL933) was isolated from raw hamburger meat linked to an outbreak of HC. Further details regarding these strains have been published previously (Mei et al., 2015). *E. coli* O157:H7 (EDL933) possesses genes encoding Stx1 and Stx2, intimin gene (*eae*) and flagellin gene (*fliC*). VTEC O26:H11 (EC20070549) and O103:H2 (EC19970811) do not have *stx2* genes. VTEC strains O111:NM (EC20070546) and O145:NM (EC19970355) do not have genes encoding Stx2 and flagellin. Enteroaggregative *E. coli* strain O104:H4 (NML#113088) does not possess genes encoding Stx1 and intimin. For simplicity, VTEC strains will be referred to by their serotype as each strain has its distinct serotype. All the strains were grown in Tryptic soy broth (TSB) at 37°C with shaking (180 rpm) for 18 h. Subsequently 0.5 mL of 18 h culture of each VTEC strain was inoculated into 50 mL pre-warmed TSB and incubated for 3 h at 37°C with shaking (180 rpm). The bacterial cells in logarithmic phase were collected, washed twice with sterile distilled water and used to inoculate lettuce samples.

### Hydrogen Peroxide Treatment of Contaminated Lettuce

Romaine lettuce was purchased from a local grocery store in Guelph. The outer leaves were removed and the remaining leaves were cut into 4 × 10 cm slices. Twenty-five grams of the leaves were weighed, placed into sterile polyethylene stomacher bags, and inoculated with 5 mL of bacterial suspension (10^8^ CFU/g). The leaves were massaged for 2 min to distribute the bacterial suspension evenly on the leaves. Subsequently, 195 mL of H_2_O_2_ was added to the bag to achieve a final concentration of 50 mM. The negative control sample contained lettuce leaves with 5 mL of autoclaved distilled water and 195 mL H_2_O_2_. The non-treated control sample contained 5 mL of bacterial suspension and 195 mL of autoclaved distilled water. The bags were incubated at room temperature for 40 min. After incubation, 1 mL of culture was taken from each stomacher bag and serially diluted with 0.1% (w/v) buffered peptone water. Subsequently, 100 μL of each dilution was spread on cefixime/tellurite—Sorbitol MacConkey agar (CT-SMAC) plates in duplicate. The plates were incubated for 18 h at 37°C, and the CFU/mL was calculated. Additionally, 4 mL samples from controls and each stomacher bag treated with 50 mM H_2_O_2_ were collected for RNA extraction.

### RNA Extraction and cDNA Synthesis

Bacterial cells were concentrated by centrifugation before RNA extraction. After taking samples from controls and each stomacher bag treated with 50 mM H_2_O_2_, each 4 mL sample was added to 8 mL (2 volume) of RNAprotect Bacteria Reagent (Qiagen). The samples were centrifuged for 10 min at 5000×*g* and supernatants were decanted prior to RNA extraction. RNA was isolated using the RNeasy Mini Kit (Qiagen, Mississauga, ON, Canada), following the manufacturer’s instructions for Gram-negative bacteria. Contaminating genomic DNA was removed from each RNA preparation using the Turbo DNA-free^TM^ kit (Ambion, Cambridge, UK), according to the manufacturer’s instructions for DNase treatments. Total RNA concentration was determined using a Thermo Scientific Nanodrop 2000 (ON, Canada). Subsequently, 0.3 μg of RNA was reverse-transcribed using SuperScript III (Invitrogen, Carlsbad, CA, USA) according to the manufacturer’s instructions. A no reverse transcriptase control (No RT) was included for each DNase-treated RNA sample.

### Real-Time PCR

All stress-associated and virulence genes tested in this study are described in **Table 1**. Transcription of key stress and virulence genes (*oxyR*, *sodA*, *soxR*, *uspA*, *rpoS*, *phoA*, *dps*, *cspA*, *cspC*, *cspE*, *gadA*, *gadB*, *gadW*, *mutS* as well as *eae*, *stx1A*, *stx2A*, and *fliC*) was evaluated using a comparative real-time PCR. Each 25-μL reaction contained 1 μL of reverse-transcribed cDNA, 12.5 μL of Power SYBR^®^ Green PCR Master Mix, 0.25 U AmpErase^®^ Uracil *N*-Glycosylase (UNG; Applied Biosystems), 2.0 μM of each primer and 6.25 μL nuclease-free water. All primers used in the study were previously described (Mei et al., 2015). Amplification and detection were carried out on a MX3500^®^ Multiplex Quantitative PCR System (Stratagene, La Jolla, CA, USA) with an initial temperature of 50°C for 2 min. Following denaturation at 95°C for 10 min, reactions were cycled 40 times as follows: amplification at 95°C for 15 s, annealing at 60°C for 30 s, extension at 72°C for 30 s. Subsequent melt curve analysis involved heating the products to 95°C for 1 min, followed by cooling to 55°C for 30 s, and heating to 95°C while monitoring fluorescence. No template control and No RT were included for each assay and no Ct values were obtained for all negative controls after 40 cycles of PCR (data not shown). Several well-known candidate reference genes including 16S rRNA, *tufA/B*, *mdh*, *pyrC*, *gatB*, *recA*, *serC*, *frr*, *rpsU*, *udp*, *mdoG*, *rpoA*, and *arcA* were tested for expression stability as previously described (Mei et al., 2015). Only 16S rRNA gene expression was stable in all VTEC strains on lettuce under experimental conditions. Therefore, 16S rRNA was used as reference gene in this study to normalize the data. Serial dilutions of the cDNA template were examined with 16S rRNA primers. Using 50-fold dilutions the Ct value was around 12, and with other primers for different target genes all the Ct values were between 20 and 35. Therefore, 50-fold dilutions of cDNA samples were used in the experiment.

**Table 1:** List of stress-associated and virulence genes tested for differential gene transcription upon treatment of VTEC strains on lettuce with hydrogen peroxide.

Stress-associated and virulence genes	Protein encoded/function
**Oxidative stress**	
*oxyR*	DNA-binding transcriptional regulator OxyR
*sodA*	Manganese-containing superoxide dismutase
*soxR*	Redox-sensitive transcriptional activator of oxidative stress regulon
**General stress**	
*uspA*	Universal stress global response regulator
*rpoS*	Regulator of the general stress response (σ^S^)
**Starvation**	
*phoA*	Alkaline phosphatase
*dps*	DNA-binding protein from starved cells
**Cold shock**	
*cspA*	Cold shock protein A
*cspC*	Cold shock protein C
*cspE*	Cold shock protein E
**UV**	
*mutS*	DNA mismatch repair protein—Mutator S
**Acid resistance**	
*gadA*	Glutamate decarboxylase A
*gadB*	Glutamate decarboxylase B
*gadW*	DNA-binding transcriptional activator GadW
**Intimin**	
eae	Intimin (adherence protein)
**Toxin**	
*stx1A*	Shiga-like toxin 1 A subunit
*stx2A*	Shiga-like toxin 2 A subunit
**Motility**	
*fliC*	Flagellin

### Statistical Analysis

The effect of H_2_O_2_ treatment on survival and gene transcription of VTEC strains present on lettuce was investigated by at least three independent experiments. Each biological sample was run in duplicate on real-time RT PCR. Relative mRNA levels were determined according to the method described by Hellemans et al. (2007). Gene transcription data were analyzed using Student’s *t*-test. Data on reduction of VTEC populations on lettuce following exposure to H_2_O_2_ were analyzed using one-way ANOVA.

## Results

Treatment of contaminated lettuce with 50 mM H_2_O_2_ for 40 min reduced the populations of all VTEC strains tested in this study by 2.4–2.8 log_10_ (**Table 2**). The differences in sensitivity to H_2_O_2_ treatment on lettuce between VTEC O157:H7 and non-O157 serotypes were not statistically significant (*P* > 0.05).

**Table 2:** Effect of H_2_O_2_ treatment on populations of six VTEC strains on lettuce.

*E. coli* strain	Mean log reduction ± SE
O157:H7 (EDL933)	2.42 ± 0.15
O26:H11 (EC20070549)	2.73 ± 0.25
O103:H2 ((EC19970811)	2.81 ± 0.15
O104:H4 (NML#11-3088)	2.53 ± 0.18
O111:NM (EC20070546)	2.82 ± 0.25
O145:NM (EC19970355)	2.52 ± 0.26

*Data are expressed as mean log reduction ± SE*.

**FIGURE 1:**
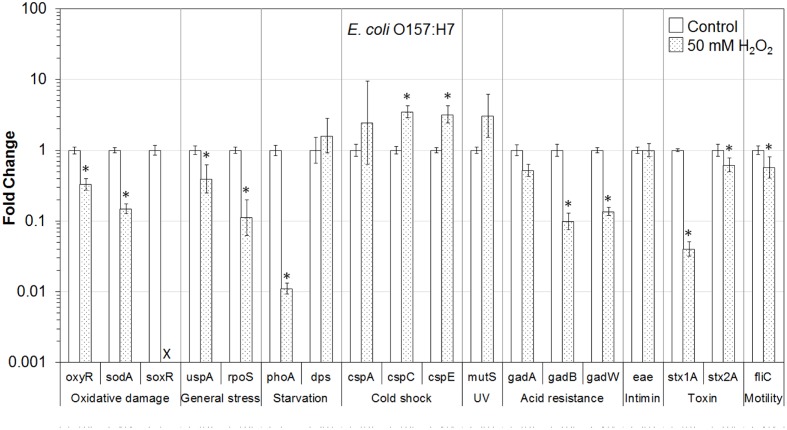
**Effect of H_2_O_2_ on gene transcription of *E. coli* O157:H7**. Relative gene transcription represents the change in transcription compared to the bacteria without H_2_O_2_ treatment (control, value of 1.0). The transcription of each gene was normalized to the 16S rRNA transcription in each sample. Data are expressed as the means ± SE for RNA extracted in three biological replicates. × denotes no transcription of *soxR* by *E. coli* O157:H7 after exposure to H_2_O_2_ on lettuce, however, *soxR* is present in this strain. ^∗^denotes the significant change of gene transcription between treatment and control.

**FIGURE 2:**
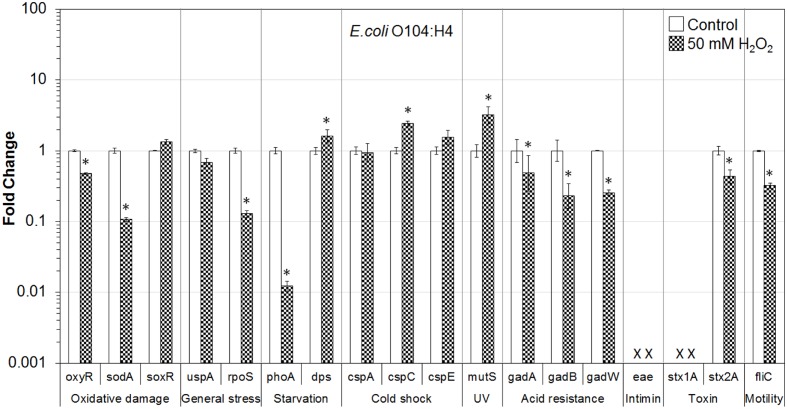
**Effect of H_2_O_2_ on gene transcription of *E. coli* O104:H4**. Relative gene transcription represents the change in transcription compared to the bacteria without H_2_O_2_ treatment (control, value of 1.0). The transcription of each gene was normalized to the 16S rRNA transcription in each sample. Data are expressed as the means ± SE for RNA extracted in three biological replicates. × denotes absence of *eae* and *stx1A* genes in *E. coli* O104:H4. ^∗^indicates the significant change of gene transcription between treatment and control.

**FIGURE 3:**
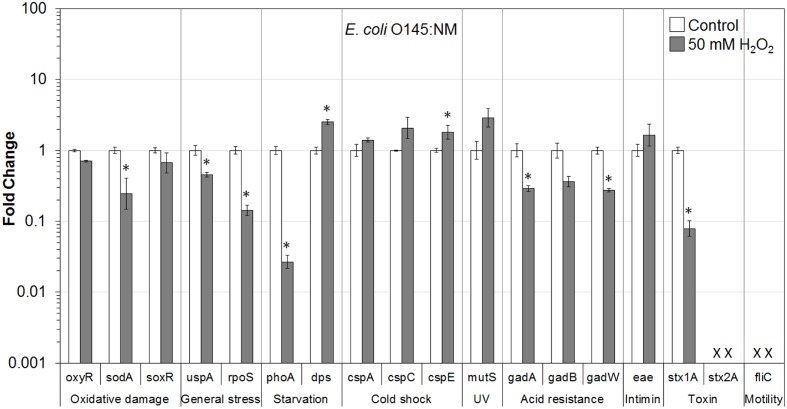
**Effect of H_2_O_2_ on gene transcription of *E. coli* O145:NM**. Relative gene transcription represents the change in transcription compared to the bacteria without H_2_O_2_ treatment (control, value of 1.0). The transcription of each gene was normalized to the 16S rRNA transcription in each sample. Data are expressed as the means ± SE for RNA extracted in three biological replicates. × denotes absence of *stx2A* and *fliC* genes in *E. coli* O145:NM. ^∗^indicates the significant change of gene transcription between treatment and control.

**FIGURE 4:**
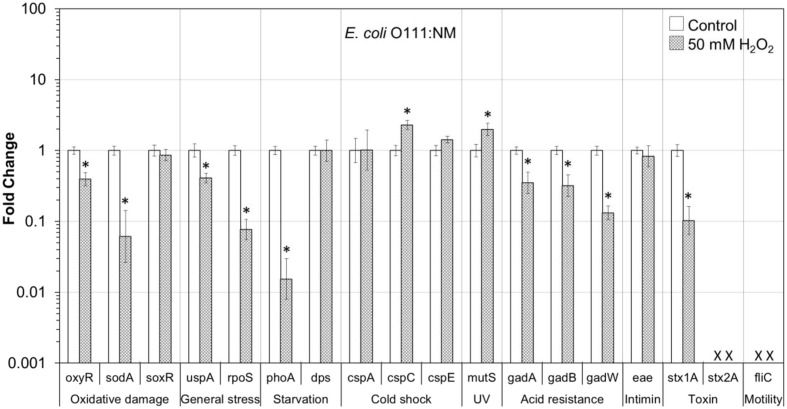
**Effect of H_2_O_2_ on gene transcription of *E. coli* O111:NM**. Relative gene transcription represents the change in transcription compared to the bacteria without H_2_O_2_ treatment (control, value of 1.0). The transcription of each gene was normalized to the 16S rRNA transcription in each sample. Data are expressed as the means ± SE for RNA extracted in three biological replicates. × denotes absence of *stx2A* and *fliC* genes in *E. coli* O111:NM. ^∗^indicates the significant change of gene transcription between treatment and control.

**FIGURE 5:**
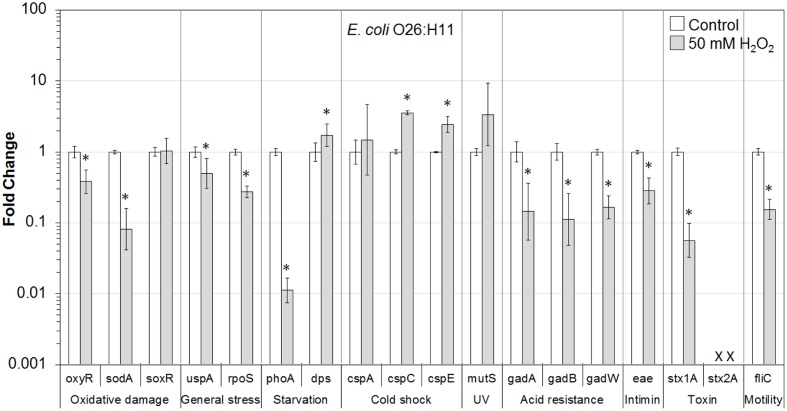
**Effect of H_2_O_2_ on gene transcription of *E. coli* O26:H11**. Relative gene transcription represents the change in transcription compared to the bacteria without H_2_O_2_ treatment (control, value of 1.0). The transcription of each gene was normalized to the 16S rRNA transcription in each sample. Data are expressed as the means ± SE for RNA extracted in three biological replicates. × denotes absence of *stx2A* gene in *E. coli* O26:H11. ^∗^indicates the significant change of gene transcription between treatment and control.

**FIGURE 6:**
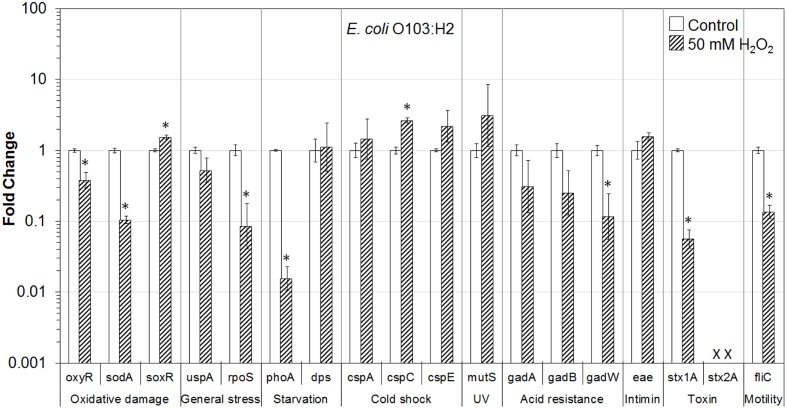
**Effect of H_2_O_2_ on gene transcription of *E. coli* O103:H2**. Relative gene transcription represents the change in transcription compared to the bacteria without H_2_O_2_ treatment (control, value of 1.0). The transcription of each gene was normalized to the 16S rRNA transcription in each sample. Data are expressed as the means ± SE for RNA extracted in three biological replicates. × denotes absence of *stx2A* gene in *E. coli* O103:H2. ^∗^indicates the significant change of gene transcription between treatment and control.

In addition, the influence of H_2_O_2_ treatment on stress and virulence gene transcription of six VTEC strains on romaine lettuce was investigated. This study focused on well-known virulence genes including genes encoding intimin (*eae*), Stx genes *stx1A* and *stx2A* and flagellin genes (*fliC*) (**Figures 1**–**6**). Transcription of key stress-associated genes such as genes involved in response to oxidative damage (*oxyR*, *sodA*, and *soxR*), general stress (*uspA* and *rpoS*), and starvation (*phoA* and *dps*) was investigated. The study also focused on the effects of H_2_O_2_ treatment of contaminated lettuce on transcription of acid stress genes (*gadA*, *gadB*, and *gadW*), cold shock (*cspA*, *cspC*, and *cspE*), and gene related to UV radiation stress (*mutS*) (**Figures 1**–**6**). A fold change cutoff of 1.5 was applied in this study.

In *E. coli* O157:H7 present on lettuce, the genes associated with oxidative stress (*oxyR* and *sodA*), universal stress (*uspA* and *rpoS*), starvation (*phoA*), acid stress (*gadB* and *gadW*), and virulence (*stx1A*, *stx2A*, and *fliC*) were significantly (*P* ≤ 0.05) downregulated after exposure to 50 mM H_2_O_2_. The *gadA* gene was downregulated 1.9-fold and the expression of *soxR* was below the detectable level. Interestingly, two genes related to cold shock (*cspC* and *cspE*) were significantly (*P* ≤ 0.05) upregulated. Genes related to starvation (*dps*), cold shock (*cspA*), and mismatch repair (MMR; *mutS*) were also upregulated (**Figure 1**).

In the case of *E. coli* O104:H4 on lettuce, H_2_O_2_ treatment caused significant (*P* ≤ 0.05) downregulation of genes related to oxidative stress (*oxyR* and *sodA*), general stress (*rpoS*), acid resistance (*gadA*, *gadB*, and *gadW*), and virulence (*stx2A* and *fliC*). The *uspA* gene was also downregulated. Transcription of *dps*, *cspC*, and *mutS* was significantly (*P* ≤ 0.05) upregulated. In addition, gene encoding cold shock protein (*cspE*) was upregulated (**Figure 2**).

In *E. coli* O145:NM present on lettuce, genes related to oxidative damage (*sodA*), general stress (*uspA* and *rpoS*), starvation (*phoA*), acid resistance (*gadA* and *gadW*), and virulence (*stx1A*) were significantly (*P* ≤ 0.05) downregulated. The *soxR* and *gadB* genes were also downregulated. Only two genes (*dps* and *cspE*) were significantly (*P* ≤ 0.05) upregulated. Furthermore, genes encoding cold shock protein (*cspC*), MMR sensor (*mutS*), and intimin (*eae*) were upregulated (**Figure 3**).

In *E. coli* O111:NM on lettuce, nine genes, responsible for oxidative damage (*oxyR* and *sodA*), general stress (*uspA* and *rpoS*), starvation (*phoA*), acid resistance (*gadA*, *gadB*, and *gadW*) and virulence (*stx1A*), were significantly (*P* ≤ 0.05) downregulated. Whereas, genes for cold shock (*cspC*) and MMR (*mutS*) were significantly (*P* ≤ 0.05) upregulated (**Figure 4**).

In *E. coli* O26:H11 present on lettuce, 9 out of 17 genes, including genes associated with oxidative damage (*oxyR* and *sodA*), general stress (*uspA* and *rpoS*), starvation (*phoA*), acid resistance (*gadA*, *gadB*, and *gadW*), and virulence (*eae*, *stx1A*, and *fliC*), were significantly (*P* ≤ 0.05) downregulated. Three genes, *dps* (starvation-related), *cspC* and *cspE* (cold shock-related), were significantly (*P* ≤ 0.05) upregulated. In addition, genes for cold shock (*cspA*) and DNA damage repair (*mutS*) were upregulated (**Figure 5**).

In *E. coli* O103:H2 on lettuce, genes related to oxidative stress (*oxyR* and *sodA*), general stress (*rpoS*), starvation (*phoA*), acid resistance (*gadW*), and virulence (*stx1A* and *fliC*) were significantly (*P* ≤ 0.05) downregulated. In addition, two genes associated with acid resistance—*gadA* and *gadB*, as well as *uspA* gene were downregulated. Interestingly, *soxR* and *cspC*, were significantly (*P* ≤ 0.05) upregulated. Genes encoding cold shock protein (*cspA* and *cspE*), and intimin (*eae*) were also upregulated (**Figure 6**).

Overall, most of the genes associated with stress and virulence were downregulated in O157:H7 and non-O157 serotypes on lettuce treated with 50 mM H_2_O_2_. Only three genes, associated with cold shock (*cspC* and *cspE*) and MMR (*mutS*), were upregulated in all VTEC strains tested (**Figures 1**–**6**).

## Discussion

The effect of H_2_O_2_ on the survival on lettuce of six VTEC strains, representing O26:H11, O103:H2, O104:H4, O111:NM, O145:NM, and O157:H7 serotypes, was investigated in this study. A population reduction of 2.4–2.8 log_10_ was observed for all VTEC strains on lettuce following treatment with 50 mM H_2_O_2_ (**Table 1**). A previous study in our laboratory, using the same VTEC strains suspended in TSB, reported greater sensitivity to H_2_O_2_. For instance treatment, of pure broth culture of VTEC O104:H4 with 1 mM H_2_O_2_ resulted in a 2.7 log reduction and 2.5 mM H_2_O_2_ caused a 3.7 log reduction in population of *E. coli* O104:H4 (Tang and Kostrzynska, 2012). Therefore, the results derived from these studies demonstrate that H_2_O_2_ treatment is less effective in lettuce decontamination compared to its effect on reducing VTEC populations in pure cultures. Previous investigations showed that several factors, such as organic loads of fresh-cut produce (Gonzalez et al., 2004), whole- or cut-leaf wash (Nou and Luo, 2010), and leaf age (Brandl and Amundson, 2008), can influence the efficacy of sanitizer to inactivate *E. coli* O157:H7 on fresh produce. These studies indicate that the lettuce leaves may help to protect bacteria, making it harder to eliminate pathogens from fresh-cut lettuce. Indeed, bacterial cells on lettuce may be physically sequestered from H_2_O_2_ exposure. In addition, organic molecules released by fresh-cut lettuce will react with H_2_O_2_ and therefore reduce the effective exposure to H_2_O_2_. Therefore, in order to choose an effective treatment to reduce the pathogens on leafy greens, it is important to understand the behavior of bacteria on lettuce under different stress conditions.

To get a better understanding of the response to H_2_O_2_ treatment in different VTEC serotypes, in the present study, the transcription of genes related to stress and virulence in O157:H7 and non-O157 serotypes on lettuce was evaluated. Different gene transcription profiles following exposure to H_2_O_2_ were observed in VTEC strains on lettuce compared to pure broth cultures (Mei et al., 2015). H_2_O_2_ caused dramatic downregulation of stress-associated and virulence genes in VTEC strains present on lettuce, including O157 and non-157 serotypes. Ten genes were downregulated in all six VTEC strains on lettuce (**Figures 1**–**6**). However, only three genes were upregulated. These genes belong to different regulation systems in *E. coli* and respond to various environmental conditions by adjusting behavior accordingly to protect cells from damaging.

### Anti-Oxidant System Response (*oxyR*, *sodA*, and *soxR*)

Superoxide dismutases (SODs) and catalases are employed by *E. coli* to respond to superoxide and peroxide stress. There are three SODs, including MnSOD (*sodA*), FeSOD (sodB), and CuZnSOD (sodC), and two catalases (HPI and HPII, encoded by *katG* and *katE*, respectively) in *E. coli*. These SODs and catalase genes are regulated by two major oxidative stress regulons, OxyR and SoxRS (Chiang and Schellhorn, 2012). In the presence of oxidative stress, OxyR can sense H_2_O_2_ and be converted to the oxidized form, subsequently activating transcription of the OxyR regulon genes, such as *katG*, *dps*, and *oxyR*, which protect the cell from H_2_O_2_ toxicity (Storz et al., 1990a; Farr and Kogoma, 1991; Chiang and Schellhorn, 2012). In this study, the transcription of *oxyR* was downregulated in all VTEC strains (**Figures 1–6**), this may cause a reduction in catalase expression, increasing peroxide sensitivity.

SoxRS is another key regulon triggered under oxidative stress in *E. coli*. It is positively regulated by superoxide-generating agents such as paraquat and constitutes a two-stage regulatory system, in which SoxR, activated as a transcriptional activator, induces the expression of *soxS* and the resulting increased levels of SoxS protein regulate the transcription of the various genes important for responding to oxidative stress (Demple and Amabile-Cuevas, 1991; Nunoshiba et al., 1992; Chiang and Schellhorn, 2012). The genes controlled by *soxRS* include *sodA* (Mn-containing SOD), *nfo* (DNA repair endonuclease IV), *micF* (antisense regulator of *ompF*), and *zwf* (glucose-6-phosphate dehydrogenase) (Christman et al., 1985; Greenberg et al., 1990; Tsaneva and Weiss, 1990). In the present study, following exposure of VTEC strains on lettuce to 50 mM H_2_O_2_, the transcription of *soxR* in *E. coli* O103:H2 slightly increased (1.5-fold), whereas, the transcription of *soxR* decreased to below the detectable level in O157:H7. Therefore, SodA reduction may be attributed to the reduction or only slight induction of soxR. Manchado et al. (2000) reported that the expression of OxyR-regulated genes (*katG* and *dps*) were induced when the concentration of H_2_O_2_ was from 1 to 100 μM, while the higher concentration (≥500 μM H_2_O_2_) resulted in the upregulation of *soxS* and *sodA*. That study suggests that the expression of *oxyR* or *soxRS* is dose-dependent. In addition, downregulation of soxR was also observed in VTEC broth cultures exposed to oxidative stress (Mei et al., 2015), however, the transcription of *sodA* was significantly (*P* ≤ 0.05) increased in pure cultures of all VTEC strains. The inconsistent results of expression of *soxR* and *sodA* may indicate that (1) sodA can react to superoxide stress independently; (2) SoxRS is not the sole regulator of *sodA* gene.

### General Stress Response (*uspA* and *rpoS*) and Cold Shock Response (*cspA*, *cspC*, and *cspE*)

*Escherichia coli* contains a large CspA family, consisting of nine homologous cold inducible proteins, CspA to CspI, among which, CspA is the major one produced at 10–24°C, and is negatively downregulated by cspC. Whereas, CspC and CspE are constitutively produced at 37°C and are not temperature regulated (Yamanaka et al., 1994; Etchegaray et al., 1996; Phadtare and Inouye, 2001). It has been reported that CspC and CspE are important regulators of the expression of RpoS, a global stress response regulator, and UspA, universal stress protein A responding to various general stresses (Nystrom and Neidhardt, 1992, 1994). In addition, RpoS-controlled genes such as *dps* and *katG* are upregulated or downregulated by the overexpression or deletion of *cspC* and *cspE* (Phadtare and Inouye, 2001; Phadtare et al., 2006). Therefore, CspC and CspE play important roles in the stress response of *E. coli*. Upregulation of *cspC* and *cspE* in all six VTEC strains on lettuce was observed in the present study (**Figures 1**–**6**). However, *uspA* and *rpoS* were downregulated, and a minor change in the transcription of *dps* was observed. Interestingly, the transcription of *cspC* was downregulated in broth cultures of all VTEC strains exposed to 2.5 mM H_2_O_2_ at 37°C (Mei et al., 2015). On the other hand, in the present study *cspC* was upregulated in VTEC strains on lettuce treated with 50 mM H_2_O_2_ at room temperature. Previous investigation showed that CspC and CspE cannot be induced under some stress conditions, like 0.5 M NaCl, 0.5 M KCl, 5% ethanol, pH 10, pH 4, temperature of 15 or 50°C (Phadtare and Inouye, 2001). It is possible that a different concentration of H_2_O_2_ used for lettuce decontamination as well as environmental factors such as H_2_O_2_ released from fresh-cut lettuce leaves as well as nutrients present in lettuce samples and natural microbiota contributed to the induction of *cspC* and *cspE*. More interestingly, contrary results of transcription of *cspC* and *cspE* observed in response to H_2_O_2_ treatment of VTEC pure cultures and in the same strains present on lettuce, suggest that VTEC may react to the same stress differently in different environments. However, it is not possible to rule out that induction of cold stress genes in present study was more of the effect of temperature change combined with peroxide treatment. Centrifugation of bacterial cells using refrigerated centrifuge, following VTEC exposure on lettuce to H_2_O_2_ could influence transcription of cold shock genes. Further studies are required to gain a better understanding of the stress conditions that affect the *cspC* and *cspE* gene transcription in VTEC present on lettuce.

### Starvation Stress Response (*phoA* and *dps*)

The gene *phoA*, encoding alkaline phosphatase, was induced under a phosphate-limited condition, but was not synthesized in normal growth medium (Han and Lee, 2006). The *phoA* is a member of *pho* regulon, regulated by a two-protein system PhoR–PhoP (Sola-Landa et al., 2003). The *phoA* was significantly downregulated in all the VTEC strains on lettuce tested in this study (**Figures 1**–**6**). Furthermore, downregulation of *phoA* was observed in most VTEC strains when pure cultures were treated with H_2_O_2_ (Mei et al., 2015). Therefore, further studies are needed to test, if H_2_O_2_ treatment changes the sensitivity of VTEC strains to phosphate-limited condition.

The H_2_O_2_ can cause lethality of the bacterial cells through several mechanisms. It has been proposed that the primary cause of cell inactivation by H_2_O_2_ or other oxidative agents is DNA damage (Storz et al., 1990b). Thus, it is important for the cells to cope with stresses by inducing the production of a variety of DNA repair enzymes as well as catalases. Glutathione also acts to protect cells from oxidative stress. In addition, Dps—the DNA binding protein from starved cells, protects cells during environmental stresses, including oxidative stress and nutritional deprivation (Almiron et al., 1992; Calhoun and Kwon, 2011). Dps protects cells from harmful oxidative radicals by DNA binding, iron storage, and by binding and oxidizing Fe ions at ferroxidase centers. Furthermore, Dps may regulate the expression of DNA repair enzymes and catalases necessary for stress resistance. Zheng et al. (2001) reported that the expression of *dps* considerably increased when the cells were exposed to 1 mM H_2_O_2_ for 10 min. However, only minor upregulation of *dps* was observed in this study for most of VTEC strains on lettuce (**Figures 1**–**6**).

### Mismatch Repair Response (*mutS*)

MutS is a member of methylation-dependent MMR system which helps to maintain chromosome stability in *E. coli*. MutS binds to the mismatches and initiates the long-patch MMR on daughter DNA strands (Modrich and Lahue, 1996; Schofield and Hsieh, 2003). MMR has also been shown to be involved in the repair of oxidative DNA damage which causes spontaneous lesion 7,8-dihydro-8-oxo-guanine (8-oxoG or GO; Michaels and Miller, 1992; Tchou and Grollman, 1993; Lu et al., 2001). Studies have shown that the amount of MutS in *E. coli* remarkably decreased when cells were in the stationary phase and under starvation stress and the expression of MutS repair protein was negatively regulated by the RpoS and Hfq global regulators (Feng et al., 1996; Tsui et al., 1997; Li et al., 2003). In the present study, *mutS* was upregulated following exposure of VTEC on lettuce to H_2_O_2_ (**Figures 1**–**6**). This upregulation may be caused by significantly downregulated expression of RpoS, which is a negative regulator of *mutS*.

### Acid Response (*gadA*, *gadB*, and *gadW*)

Food-borne pathogenic *E. coli* must be able to survive in the extremely acidic environment of the stomach and resist very low pH (1.5–3.0) for several hours (Peterson et al., 1989). Previous studies have shown that a total of 12 genes comprise an acid fitness island, including a glutamate decarboxylase (GAD) system and three transcriptional regulators (GadE, GadX, and GadW) of the GAD enzymes, which renders *E. coli* the ability to survive strong acidic stress (Hommais et al., 2001, 2004; Ma et al., 2002; Bergholz et al., 2007; Mates et al., 2007). *E. coli* produce two isozymes of GAD encoded by the *gadA* and *gadB* genes, which are induced by GadX at any pH, while GadW represses expression of *gadX*. GadW activates the expression of *gadA* and *gadB* only in the absence of GadX (Ma et al., 2002; Tucker et al., 2003). In addition, it has been reported that the expression of *gadA* and *gadBC* were induced when bacteria were cultured in acidified medium, treated with acetate, and during entry into stationary phase (Selinger et al., 2000; Arnold et al., 2001; Tucker et al., 2002). However, the expression of regulators GadX and GadW, under the same conditions, were unknown. In the present study, *gadA*, *gadB*, and *gadW* were significantly downregulated in all six VTEC strains on lettuce (**Figures 1**–**6**). We suspect that the expression of *gadX* was also downregulated in response to H_2_O_2_, which subsequently resulted in the downregulation of *gadA* and *gadB*. Furthermore, the results indicate that following H_2_O_2_ treatment, VTEC on lettuce may become more sensitive to acid stress, which decreases the viability of pathogens under low pH.

### Virulence Factors (Encoded by *eae*, *stx1A*, *stx2A*, and *fliC* Genes)

Production of Stx is the definitive virulence factor of VTEC O157:H7 and non-O157 serotypes (Croxen et al., 2013; Scheutz, 2014; Smith et al., 2014; Franz et al., 2015). Stx produced by VTEC can be classified into two types Stx1 and Stx2. There are three subtypes of Stx1 (a, c, d) and seven subtypes of Stx2 (a, b, c, d, e, f, g). Although both toxins could cause bloody diarrhea and HUS, a specific subset of *stx2* subtypes (*stx2a*, *stx2c*, and *stx2d*) have a higher association with HC and HUS than *stx1* subtypes or other *stx2* subtypes (Scheutz, 2014). Stx are encoded on bacteriophage genomes that are integrated into bacterial chromosome. As such, the biology of the Stx-encoding phages influences the expression of *stx1* and *stx2*. Production and release of toxins depend on induction of these bacteriophages (Croxen et al., 2013; Scheutz, 2014). Amongst the VTEC strains tested in this study, *E. coli* O157:H7 produced both Stx1 and Stx2, *E. coli* O104:H4 produced only Stx2, and the remaining four strains produced only Stx1. In this study, both *stx1A* and *stx2A* genes were dramatically downregulated in all VTEC strains on lettuce following exposure to H_2_O_2_ (**Figures 1**–**6**).

Many VTEC strains produce attaching and effacing (AE) lesions, which are controlled by the locus of enterocyte effacement (LEE; Croxen et al., 2013; Scheutz, 2014). Although AE lesions are not essential for bloody diarrhea and HUS, the majority of strains implicated in these syndromes are LEE-positive. Intimin, encoded by *eae* (*E. coli* attaching and effacing) gene, is responsible for intimate adhesion of VTEC to the intestinal epithelium and formation of AE lesions. Intimin binds to the cell receptor Tir, which is translocated by the pathogen to the enterocyte via a type III secretion system (Croxen et al., 2013). The presence of *eae* gene is strongly correlated to the presence of genes encoding many other virulence factors (Franz et al., 2015). In addition, VTEC strains with *eae* and *stx2* genes have been associated with HUS and bloody diarrhea (Scheutz, 2014). In the present study, the transcription of *eae* (except for *E. coli* O104:H4 which is LEE-negative) changed only slightly following H_2_O_2_ treatment (**Figures 1**–**6**). Thus, further studies about *eae* regulation and the intimin-related virulence of VTEC strains under different stress conditions are needed.

Production of flagella is another contributor to the pathogenicity of VTEC (Gyles, 2007). Flagella are mainly responsible for motility, chemotaxis, and secretion of virulence factors. The key structural component of the flagellum filament is encoded by the *fliC* gene. In our studies, the transcription of *fliC* was dramatically downregulated in all four motile VTEC strains on lettuce treated with H_2_O_2_ (**Figures 1**–**6**). In addition, *fliC* was downregulated in pure broth cultures exposed to H_2_O_2_ (Mei et al., 2015). These results suggest that H_2_O_2_ treatment affects motility of VTEC strains.

## Conclusion

H_2_O_2_ treatment caused less dramatic reduction in VTEC populations on lettuce compared to pure broth cultures, which indicates that VTEC are protected from H_2_O_2_ on leafy greens. Consequently higher concentration of H_2_O_2_ or other sanitizers are required to reduce and eliminate VTEC on lettuce. As such, the results derived from this study showed the importance of food matrix in studying the effect of sanitizers on survival rate of VTEC. In addition, VTEC strains on lettuce showed similar transcription patterns (regardless of serotype) in response to treatment with 50 mM H_2_O_2._ The transcription of genes related to oxidative stress, general stress, acid stress and some virulence genes were significantly downregulated in six VTEC serotypes. Consequently, VTEC strains on lettuce may become more sensitive to acid stress following H_2_O_2_ treatment. Interestingly, different gene transcription patterns in response to H_2_O_2_ were observed in VTEC strains on lettuce compared to pure broth cultures (Mei et al., 2015). Particularly, *sodA* gene encoding manganese SOD was significantly upregulated in pure cultures of all VTEC serotypes, however, this gene was downregulated in the same strains present on lettuce. In addition, Stx genes were upregulated in broth culture of *E. coli* O157:H7 and downregulated in most VTEC serotypes (including O157:H7) on lettuce following H_2_O_2_ treatment. It is possible that factors released from fresh-cut lettuce leaves and natural microbiota present in lettuce samples contributed to different gene transcription patterns in VTEC strains on lettuce compared to broth cultures.

## Author Contributions

All authors have made substantial direct and intellectual contributions to this work.

## Conflict of Interest Statement

The authors declare that the research was conducted in the absence of any commercial or financial relationships that could be construed as a potential conflict of interest.
